# Membrane Protein OTOF Is a Type I Interferon-Induced Entry Inhibitor of HIV-1 in Macrophages

**DOI:** 10.1128/mbio.01738-22

**Published:** 2022-07-18

**Authors:** Haibo Ding, Xiaowei Zhang, Zheming Zhu, Shumei Wang, Ying Xiong, Hong Shang, Guoxin Liang

**Affiliations:** a Key Laboratory of AIDS Immunology of Ministry of Health, Department of Laboratory Medicine, The First Affiliated Hospital, China Medical University, Shenyang, China; b Research Institute for Cancer Therapy, The First Affiliated Hospital, China Medical University, Shenyang, China; c National Clinical Research Center for Laboratory Medicine, The First Affiliated Hospital, China Medical University, Shenyang, China; d Collaborative Innovation Center for Diagnosis and Treatment of Infectious Diseases, Hangzhou, China; Columbia University Medical Center

**Keywords:** HIV-1, OTOF, interferons

## Abstract

In humans, HIV-1 infection induces innate immune responses mediated mainly by type I interferon (IFN). Type I IFN restricts HIV-1 replication by upregulating the expression of IFN-stimulated genes with diverse anti-HIV properties. In this study, we report that the cell membrane protein otoferlin (OTOF) acts as a type I IFN-induced effector, inhibiting HIV-1 entry in myeloid lineage macrophages and dendritic cells (DCs). OTOF is significantly induced by type I IFN in macrophages and DCs but not in CD4^+^ T lymphocytes. Silencing *OTOF* abrogates the IFN-mediated suppression of HIV-1 infection in macrophages and DCs. Moreover, OTOF overexpression exhibits anti-HIV activity in macrophages and CD4^+^ T cells. Further evidence reveals that OTOF inhibits HIV-1 entry into target cells at the cell membrane. Collectively, OTOF is a downstream molecule induced by type I IFN to inhibit HIV-1 entry in macrophages; it is a new potential agent for the treatment of HIV infection.

## INTRODUCTION

HIV-1 infects several types of immune cells. Myeloid lineage cells, including macrophages and dendritic cells (DCs), as well as CD4^+^ T lymphocytes are the major targets of HIV-1 *in vivo* (1). Both macrophages and DCs function as vehicles for virus dissemination through the body and also as viral reservoirs *in vivo* (1–4). Restriction factors, which block specific steps in the replication cycle of HIV (5–10) and are inducible by interferons (IFNs), are a major component of host anti-HIV innate immunity. HIV-1 infection induces the production of type I IFN, which subsequently upregulates the expression of IFN-stimulated genes (ISGs). Multiple ISGs have been reported to inhibit HIV-1 replication at different steps of the viral life cycle through various mechanisms (11–19). Extensive *in vitro* studies using cell culture systems have elucidated that certain ISGs such as IFN-induced transmembrane proteins (IFITMs), cholesterol-25-hydroxylase (CH125h), APOBEC3G, tetherin, SAM and HD domain-containing protein 1 (SAMHD1), MX2, and Shiftless exert robust anti-HIV-1 activity (18–29), suggesting that type I IFN responses triggered by HIV-1 infection are detrimental to viral spread. When HIV-1 infects human immune cells, pattern recognition receptors and cytosolic sensors engage in the detection of viral cDNA and RNA, respectively. Then, viral cDNA is identified by IFN-inducible protein 16 (IFI16) or cyclic GMP–AMP (cGAMP) synthase. Subsequently, type I IFN is produced in target cells via IFI16 and the cGAMP-activating stimulator of the IFN gene *STING* (30–33).

In this study, by comparing the gene expression profiles in peripheral blood mononuclear cells (PBMCs) isolated from untreated patients with HIV-1 infection and healthy donors, we discovered that a transmembrane protein, otoferlin (OTOF) (34–37), acts as an IFN-α-induced inhibitor of HIV-1 infection in macrophages and DCs. IFN-α was observed to significantly induce *OTOF* expression in macrophages, DCs, or propidium monoazide (PMA)-treated THP-1 cells but only slightly induce it in primary CD4^+^ T lymphocytes. Interestingly, *OTOF* overexpression reduces host susceptibility to HIV-1 and HIV-2 but not equine infectious anemia virus (EIAV) or murine leukemia virus (MLV). Moreover, the depletion of *OTOF* using RNA interference (RNAi) reduces the anti-HIV-1 potency of IFN-α in macrophages and DCs. Further evidence suggested that OTOF suppresses HIV-1 entry into target cells, and this inhibitory effect remained unaffected by the additional treatment with Ca^2+^. Overall, these findings collectively indicate that *OTOF* is an effector of the anti-HIV-1 activity of type I IFN in macrophages and DCs, suggesting a new potential anti-HIV-1 strategy.

## RESULTS

### IFN-α induces high OTOF expression in macrophages and DCs.

**FIG 1:**
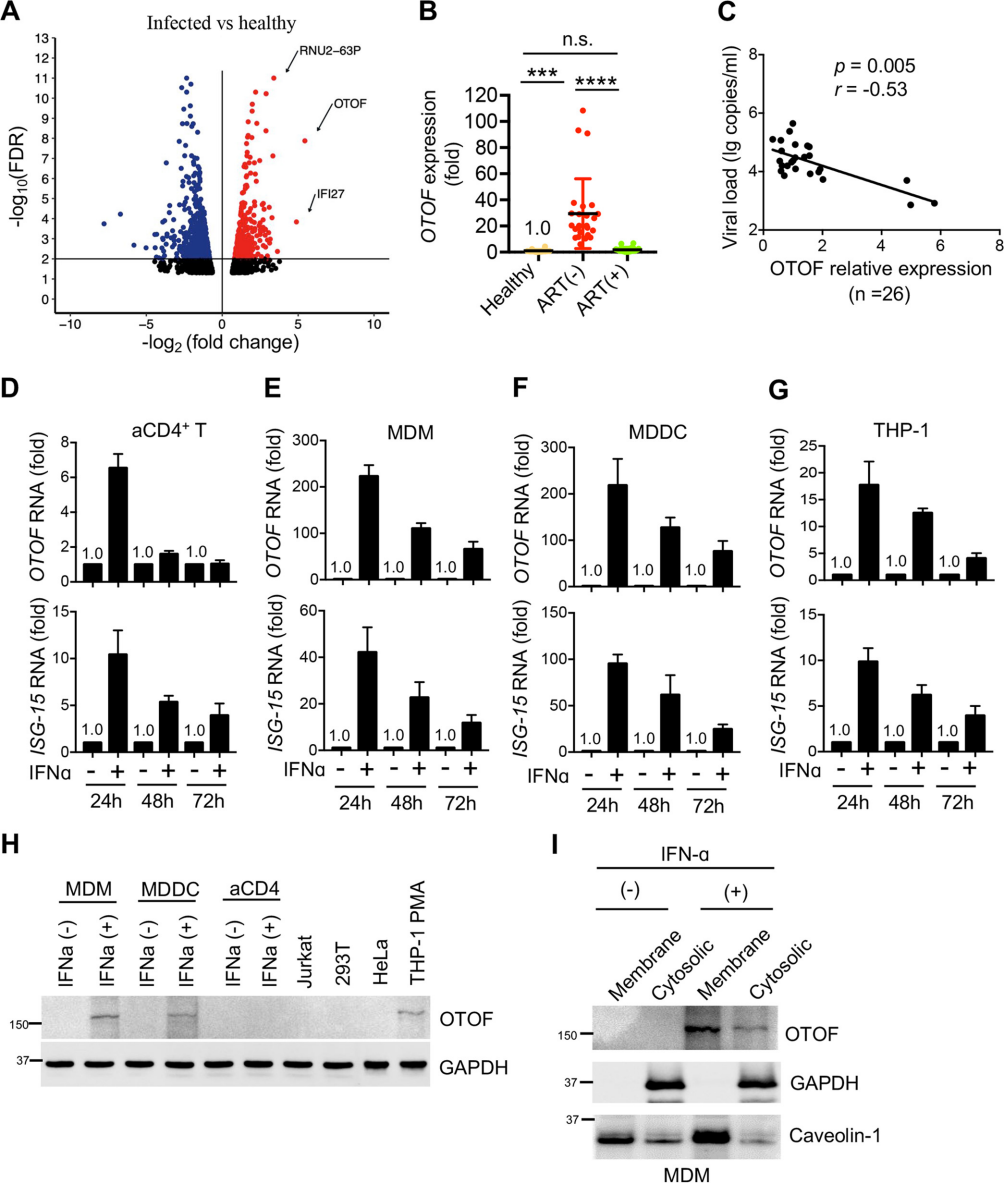
IFN-α treatment induces *OTOF* in macrophages. (A) *OTOF* was induced in the PBMCs of untreated patients with HIV infection. Volcano plot of genes differentially expressed in PBMCs isolated from five independent healthy donors and five independent untreated patients with HIV-1 infection. The log_2_ fold change difference is presented on the *x* axis, and the negative log of the false-discovery rate (FDR) is presented on the *y* axis. Each point represents one gene, which had detectable expression in both groups (five untreated patients with HIV-1 infection versus five healthy donors). The significantly differentially expressed genes are plotted in red with upregulated genes on the right side and downregulated genes on the left side. The nonsignificantly differentially expressed genes are shown as black points. (B) Relative expression of *OTOF* in the PBMCs of healthy donors (*n* = 16), patients with HIV-1 who did not receive ART (*n* = 26), and patients with HIV-1 who received ART (*n* = 20; viral loads of <50 copies/mL). Total RNA was extracted from PBMCs for quantitative PCR to measure *OTOF* transcripts normalized to *GAPDH* levels. ****, *P* < 0.0001; ***, *P* < 0.001; and n.s., not significant (two-tailed, unpaired Student’s *t* test). Error bars indicate the standard errors of the means. (C) Correlation between relative *OTOF* expression (the result of OTOF derived from one patient as “1”) in PBMCs and viral loads from untreated patients indicated in panel B. Spearman correlation was used for analysis. (D to G) Activated CD4^+^ T (aCD4) cells (D), MDMs (E), MDDCs (F), and THP-1 cells (G) were treated with IFN-α (1,000 U/mL) or left untreated for 24, 48, and 72 h. Total RNA was extracted for quantitative PCR to measure the transcript levels of *OTOF* and *ISG15* normalized to *GAPDH* levels. Data are plotted as the means ± standard deviations for three triplicates and are representative of three independent experiments. (H) Primary cells or cell lines were treated with IFN-α (1,000 U/mL) or left untreated for 24 h and then lysed for Western blotting to assess OTOF and GAPDH levels using their specific antibodies. (I) OTOF is a membrane-localized protein. MDMs were treated with IFN-α (1,000 U/mL) or left untreated for 24 h; membrane-bound and cytosolic proteins were separated to assess the levels of OTOF, caveolin-1, and GAPDH using Western blotting with specific antibodies. All Western blotting data are representative of the three independent experiments. Numbers at left of panels H and I are molecular masses in kilodaltons.

To investigate the potential factors involved in HIV-1 infection, we performed high-throughput transcriptome sequencing (RNA-Seq) using freshly isolated PBMCs from five independent untreated patients with HIV-1 infection and five healthy donors and compared their gene expression profiles. *Otoferlin* (*OTOF*) (34–37) was found to be the most strongly induced gene in a genome-wide analysis of transcripts (Fig. 1A and see Table S1 in the supplemental material). OTOF belongs to the ferlin family of multiple C2 domain proteins with emerging roles as the regulators of vesicle fusion and receptor trafficking (38). Ferlins are tail‐anchored transmembrane proteins with approximately 15 to 20 extracellular amino acids extending beyond the predicted transmembrane helix. Type I ferlins (dysferlin, myoferlin, and Fer1L5) have FerA and DysF domains, whereas type II ferlins (otoferlin, Fer1L4, and Fer1L6) do not have these domains. Ferlins other than OTOF were not upregulated in the PBMCs of untreated patients. OTOF is a type II ferlin, and it reportedly localizes in ribbon-associated synaptic vesicles, exhibiting Ca^2+^-dependent interactions with the SNARE proteins (34). OTOF acts as the major Ca^2+^ sensor, inducing membrane fusion at the auditory hair cell ribbon synapse. *OTOF* deficiency causes nonsyndromic prelingual deafness in humans, and *OTOF*-deficient mice are profoundly deaf. Our RNA-Seq data revealed that *OTOF* was significantly induced in the PBMCs of all five untreated patients *in vivo*, whereas it remained undetected in the PBMCs of healthy donors (Fig. S1A). To confirm this result, we further examined *OTOF* expression in the patients who did not receive antiretroviral therapy (ART) (Table S2), patients who received ART (Table S3), and healthy donors. We consistently observed that *OTOF* expression increased by nearly 30-fold in the PBMCs of untreated patients compared with those of ART-treated patients or healthy donors (Fig. 1B). In contrast, *OTOF* transcript levels were similar between ART-treated patients and healthy donors, indicating that *OTOF* upregulation is due to the spread of HIV-1 infection *in vivo*.

10.1128/mbio.01738-22.1 FIG S1 *OTOF* is induced in PBMCs in untreated patients with HIV infection. (A) Total RNA was extracted from PBMCs isolated from untreated patients or healthy donors and subjected to high-throughput sequencing. Integrative Genomics Viewer was used to visualize the transcript peaks of *OTOF* on the *y* axis. The maximum of the *y* axis was set as 100 to compare all samples, and the *x* axis presents all of the exons and introns of the *OTOF* locus. (B) 293T and Jurkat cells were treated with or without IFN-α (1,000 U/mL) for 24 h. Total RNA was extracted for quantitative PCR to measure the transcript levels of *OTOF* and *ISG15* normalized to *GAPDH* levels. Data are plotted as the means ± standard deviations from three triplicates and are representative of three independent experiments. (B) 293T, Jurkat, and PMA-stimulated THP-1 cells were treated with IFN-α (1,000 U/mL) or left untreated for 24 h and then lysed for Western blotting to assess OTOF and GAPDH protein levels using specific antibodies. All Western blot data are representative of three independent experiments. Download FIG S1, PDF file, 0.2 MB. Copyright © 2022 Ding et al. 2022 Ding et al. https://creativecommons.org/licenses/by/4.0/ This content is distributed under the terms of the Creative Commons Attribution 4.0 International license.

10.1128/mbio.01738-22.7 TABLE S1 Fold changes (>10-fold) in the comparison of the gene expression profiles of untreated patients and healthy donors. Download Table S1, PDF file, 0.01 MB. Copyright © 2022 Ding et al. 2022 Ding et al. https://creativecommons.org/licenses/by/4.0/ This content is distributed under the terms of the Creative Commons Attribution 4.0 International license.

10.1128/mbio.01738-22.8 TABLE S2 Background of patients with HIV-1 infection who did not receive ART. Download Table S2, PDF file, 0.01 MB. Copyright © 2022 Ding et al. 2022 Ding et al. https://creativecommons.org/licenses/by/4.0/ This content is distributed under the terms of the Creative Commons Attribution 4.0 International license.

10.1128/mbio.01738-22.9 TABLE S3 Background of patients with HIV-1 infection who received ART. Download Table S3, PDF file, 0.01 MB. Copyright © 2022 Ding et al. 2022 Ding et al. https://creativecommons.org/licenses/by/4.0/ This content is distributed under the terms of the Creative Commons Attribution 4.0 International license.

Next, we examined whether the upregulation of *OTOF* in these untreated patients was related to viral loads. *OTOF* expression was found to inversely correlate with viral loads *in vivo* (Fig. 1C), suggesting a potential inhibitory effect of *OTOF* on HIV-1 replication.

Afterward, we investigated why *OTOF* is significantly induced in patients with HIV-1 infection. As depicted in Fig. 1A, the expression of IFN-α-inducible protein 27 (IFI27; fold change, 29.6) was increased in untreated patients, and its expression level was slightly lower than that of *OTOF* (Table S1). Interestingly, we observed upregulation of the IFN-induced antiviral protein IFITM3 only in the IFITM family (fold change, 7.82; see Table S5 at https://data.mendeley.com/datasets/tvw6wdw9bz/3) in the PBMCs of untreated patients (27, 29). Thus, we hypothesized that this upregulation of *OTOF* expression might be because of IFN-α stimulation *in vivo* due to HIV-1 infection. To address this issue, we first treated primary stimulated CD4^+^ T lymphocytes with IFN-α for different periods to examine *OTOF* expression. IFN-α increased *OTOF* expression by 6-fold only during the 24-h treatment period (Fig. 1D). Considering that PBMCs contain many different immune cells and that myeloid cell lineage macrophages are crucial *in vivo* targets in patients with HIV-1 infection, we next treated primary monocyte-derived macrophages (MDMs) and monocyte-derived dendritic cells (MDDCs) with IFN-α for different periods. IFN-α significantly induced *OTOF* expression by nearly 200-fold within the 24-h treatment period (Fig. 1E and F) compared with the 40-fold induction by IFN-stimulated protein (ISG-15). However, OTOF was not highly induced in 293T and Jurkat cells in the presence of IFN-α (Fig. S1B). Thus, this striking upregulation of *OTOF* indicates that OTOF is a downstream factor induced by IFN-α in myeloid cells. To validate this assumption, we treated the stimulated THP-1 cells with IFN-α and observed that IFN-α consistently induced the cells to express high levels of *OTOF* (Fig. 1G). Notably, PBMCs have fewer primary macrophages; therefore, the high *OTOF* induction in the PBMCs of untreated patients is probably the result of multiple immune cells, including CD4^+^ T cells and DCs.

### OTOF is localized on the cell membrane.

Before exploring the function of OTOF, we examined OTOF protein levels in various HIV host cells in the presence or absence of IFN-α treatment (Fig. 1H and Fig. S1C). Data consistently indicate that OTOF protein was induced in IFN-α-stimulated macrophages, DCs, and THP-1 cells, but we did not observe sufficient protein levels in CD4^+^ T, Jurkat, 293T, or HeLa cells. Although IFN-α-stimulated CD4^+^ T cells express *OTOF* transcripts to some extent, they are probably insufficient for Western blot analysis. Therefore, we propose that OTOF is an IFN-induced protein in myeloid lineage macrophages and DCs *in vitro*. Because OTOF has been reported as a cell membrane protein (34–37), we examined the localization of OTOF in primary macrophages and consistently observed that it was located on the cell membrane of macrophages in a manner similar to that noted in the case of caveolin-1, which is a bona fide integrated plasma membrane protein (Fig. 1I). Therefore, OTOF is an IFN-induced membrane-localized protein in primary macrophages.

### IFN-α induces OTOF to restrict HIV-1 infection in myeloid cells.

**FIG 2:**
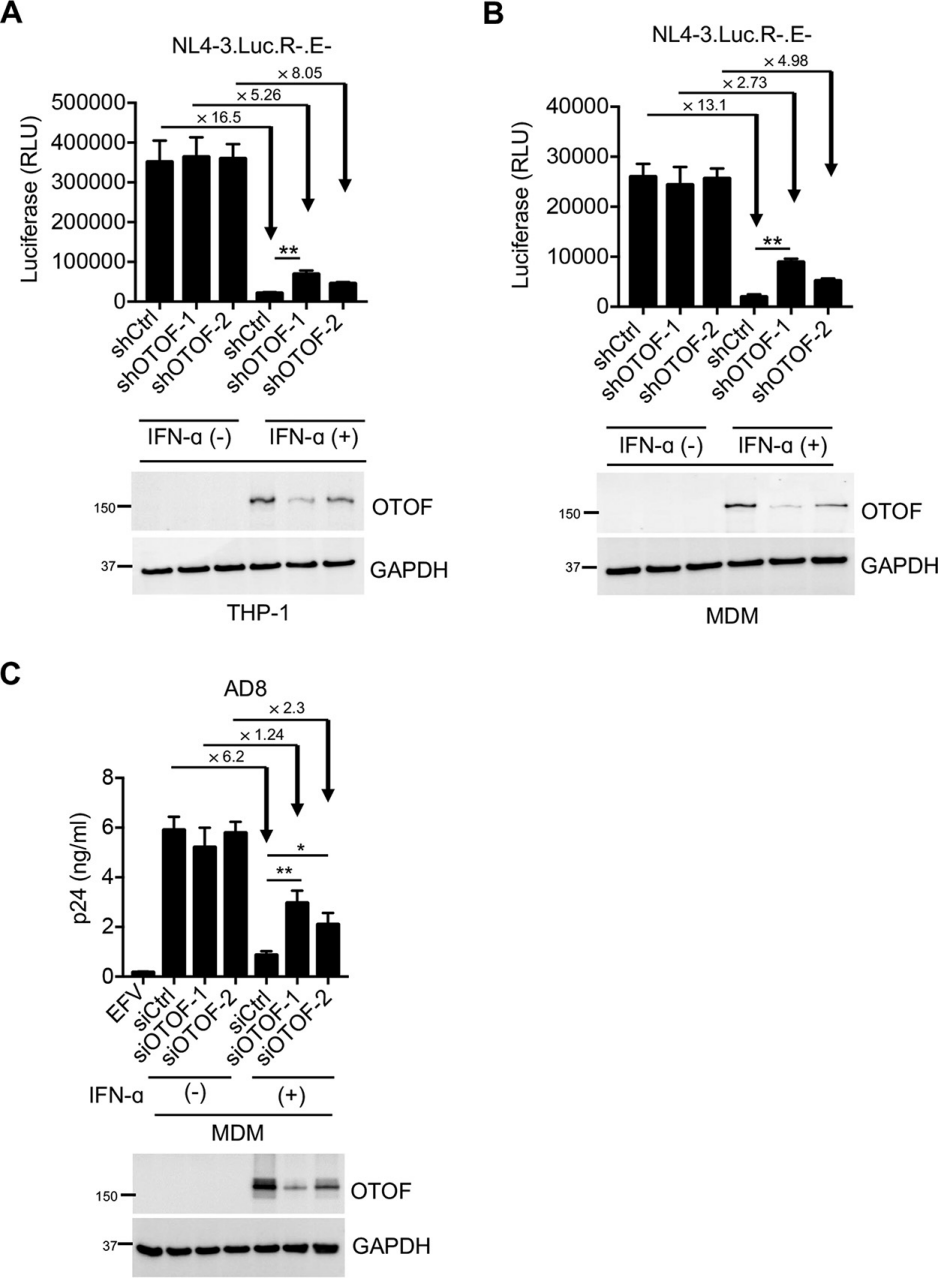
IFN-α induces OTOF to restrict HIV-1 infection in macrophages. (A and B) Lentiviral shRNA-transduced THP-1 cells (A) and MDMs (B) were pretreated with IFN-α (1,000 U/mL). At 24 h after treatment, cells were washed using culture medium and subsequently infected with 100 ng of HIV-1_NL4-3.Luc.R−.E−_ (VSV-G). At 24 h after infection, cells were lysed to measure luciferase reporter activity and for Western blotting to assess the levels of OTOF and GAPDH. RLU, relative luminescence units. (C) MDMs were transfected with siRNA against *OTOF* or control siRNA, and cells were treated with IFN-α (1,000 U/mL) 6 h after transfection. At 24 h after treatment, cells were washed and further infected with 50 ng of replication-competent HIV-1_AD8_. At 72 h after infection, viral load in culture supernatants was measured via p24 ELISA; cells were lysed for Western blotting to assess the levels of OTOF and GAPDH. *, *P* < 0.05; **, *P* < 0.01 (two-tailed, unpaired Student’s *t* test). EFV, efavirenz. Data are plotted as the means ± standard deviations for three triplicates and representative of three independent experiments. All Western blot data are representative of three independent experiments. Numbers at left of blots are molecular masses in kilodaltons.

To investigate whether IFN-α induces OTOF to suppress HIV-1 infection in myeloid cells, we silenced *OTOF* in stimulated THP-1 cells using RNAi with or without IFN-α treatment. The use of short hairpin RNA (shRNA) to deplete OTOF compromised the IFN-α-mediated resistance to HIV-1 infection (Fig. 2A). The inhibitory effect of IFN-α on HIV-1 infection dropped from 16.5- to 8-fold when OTOF was depleted. To confirm these results, we transduced lentiviral shRNA vectors into primary macrophages in the presence or absence of IFN-α and then infected these cells with a vesicular stomatitis virus G protein (VSV-G) pseudotyped HIV-1_NL4-3.Luc.R−.E−_ reporter virus. In the presence of OTOF (Fig. 2B), IFN-α treatment reduced HIV-1 infection by 13.1-fold; however, silencing *OTOF* reduced the IFN-α-mediated suppression by 2.73-fold, indicating that OTOF contributes to IFN-α-mediated resistance to HIV-1 infection in primary macrophages. Similarly, small interfering RNA (siRNA) was used to deplete OTOF induced by IFN-α, and we consistently observed that OTOF knockdown impaired the IFN-α-mediated suppression of HIV-1 infection in macrophages (Fig. S2A); however, OTOF knockdown exerted no effects on IFN-induced *ISG-15* expression, indicating that OTOF depletion did not affect cellular response to IFN. Notably, IFITM3 is also an IFN-induced antiviral protein that blocks HIV-1 virion fusion with the cell membrane. We, therefore, investigated IFITM3 protein levels in the presence or absence of OTOF (Fig. S2B). The depletion of OTOF did not influence IFN-induced IFITM3 expression, indicating that the anti-HIV activity of OTOF is not associated with IFITM3. Moreover, we evaluated the effects of OTOF on replication-competent HIV-1; the data show that OTOF consistently contributed to IFN-mediated resistance to HIV-1 infection in macrophages (Fig. 2C). Collectively, OTOF represents an IFN-α-induced anti-HIV-1 factor in macrophages.

10.1128/mbio.01738-22.2 FIG S2 IFN-α induces OTOF to restrict HIV-1 infection in macrophages and DCs. (A) MDMs were transfected with siRNA against *OTOF* or control siRNA, and cells were treated with IFN-α (1,000 U/mL) 6 h after transfection. At 24 h after treatment, cells were washed and further infected with 50 ng of HIV-1_NL4-3.Luc.R−.E−_ (VSV-G). Cells were lysed to measure luciferase reporter activity 24 h after infection. Total RNA was extracted for quantitative PCR (qPCR) to measure the levels of *OTOF* or *ISG-15* transcripts normalized to *GAPDH* levels. (B) MDMs were transfected with siRNA against *OTOF* or control siRNA, and cells were treated with IFN-α (1,000 U/mL) 6 h after transfection. At 24 h after treatment, cells were lysed for Western blotting to measure OTOF, IFITM3, and GAPDH levels using specific antibodies. (C) MDDCs were transfected with siRNA against *OTOF* or control siRNA; at 6 h after transfection, cells were treated with IFN-α (1,000 U/mL). At 24 h after treatment, cells were pretreated with VLP-Vpx and further infected with 100 ng of HIV-1_NL4-3.Luc.R−.E−_ (VSV-G). At 24 h after infection, cells were lysed to measure luciferase reporter activity. Total RNA was extracted for quantitative PCR to measure the levels of *OTOF* or *ISG-15* transcripts normalized to *GAPDH* levels. **, *P* < 0.01; *, *P* < 0.05, n.s., not significant (two-tailed, unpaired Student’s *t* test). Data are plotted as the means ± standard deviations from three triplicates and are representative of three independent experiments. All Western blot data are representative of three independent experiments. Download FIG S2, PDF file, 0.3 MB. Copyright © 2022 Ding et al. 2022 Ding et al. https://creativecommons.org/licenses/by/4.0/ This content is distributed under the terms of the Creative Commons Attribution 4.0 International license.

Because IFN-α promotes OTOF expression in MDDCs, we also wanted to explore whether this upregulated OTOF still exhibits anti-HIV-1 activity. As depicted in Fig. S2C, siRNA-mediated depletion of OTOF also restored HIV-1 infection from IFN-α-mediated suppression but exerted no effects on the IFN-induced *ISG-15* expression. IFN-α treatment reduced HIV-1 infection by 20-fold in the presence of OTOF, whereas it reduced HIV-1 infection by 6.7- to 9.4-fold in the absence of OTOF. Taken together, the membrane protein OTOF is an IFN-induced anti-HIV-1 factor in either macrophages or DCs.

### OTOF overexpression restricts HIV-1 infection.

**FIG 3:**
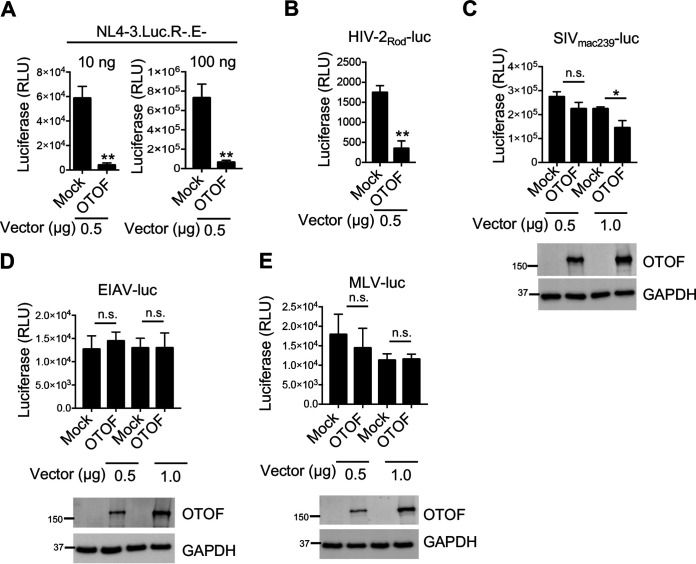
OTOF suppresses HIV-1 and HIV-2 infection and slightly suppresses SIV infection but does not suppress EIAV and MLV infection. (A) 293T cells were transfected with a construct encoding FLAG-tagged OTOF or a mock expression construct at the indicated doses. At 24 h after transfection, cells were infected with 10 or 100 ng of HIV-1_NL4-3.Luc.R−.E−_ (VSV-G). At 24 h after infection, cells were lysed to measure luciferase reporter activity. (B to E) 293T cells were transfected with a construct encoding FLAG-tagged OTOF or a mock expression construct at the indicated doses. At 24 h after transfection, cells were infected with VSV-G pseudotyped luciferase reporter viruses: HIV-2_Rod_-luc (B), SIV_mac239-_luc (C), EIAV-luc (D), or MLV-luc (E). At 24 h after infection, cells were lysed to measure luciferase reporter activity and for Western blotting to assess the levels of OTOF and GAPDH using specific antibodies. **, *P* < 0.01; *, *P *< 0.015; n.s., not significant (two-tailed, unpaired Student’s *t* test). Data are plotted as the means ± standard errors of the means for three independent experiments. All Western blot data are representative of three independent experiments. Numbers at left of blots are molecular masses in kilodaltons.

To further investigate the molecular mechanisms through which OTOF inhibits HIV-1 infection, we infected 293T cells with an HIV-1_NL4-3.Luc.R−.E−_ reporter virus at different doses in the presence or absence of overexpressed OTOF protein. The ectopic expression of OTOF resulted in significant inhibition of HIV-1 infection (Fig. 3A). In contrast, OTOF did not affect HIV-1 progeny infectivity in producer cells when we used the same OTOF expression vectors (Fig. S3A), which indicates that OTOF exerts no effects on HIV-1 infection in producer cells but may restrict HIV-1 infection in target cells. Moreover, we used HIV-1_NL4-3.Luc.R−.E−_ combined with X4-, R5-, or dual-tropic envelope (Env) glycoprotein or VSV-G to infect 293T cells expressing CD4, CXCR4, and CCR5 proteins in the presence or absence of OTOF overexpression. OTOF inhibited all infections in target cells (Fig. S3B), indicating that the OTOF-mediated suppression of HIV-1 is independent of viral glycoproteins.

10.1128/mbio.01738-22.3 FIG S3 OTOF does not affect HIV-1 infectivity in producer cells. (A) 293T cells were cotransfected with FLAG-tagged OTOF or mock expression constructs and proviral vector NL4-3. At 2 days after cotransfection, 100 ng of the produced virions in the supernatants was used to infect TZM-bl reporter cells to measure virion infectivity. Data are plotted as the means ± standard errors of the means from three independent experiments. (B) 293T cells expressing CD4, CXCR4, and CCR5 were transfected with FLAG-tagged OTOF or mock expression constructs. At 24 h after transfection, cells were infected with HIV-1_NL4-3.Luc.R−.E−_ with X4-tropic, R5-tropic, or dual-tropic Env or VSV-G, as indicated. Cells were lysed to measure luciferase activity 24 h after infection. **, *P* < 0.01; *, *P* < 0.05 (two-tailed, unpaired Student’s *t* test). Data are plotted as the means ± standard errors of the means from three independent experiments. All Western blot data are representative of three independent experiments. Download FIG S3, PDF file, 0.1 MB. Copyright © 2022 Ding et al. 2022 Ding et al. https://creativecommons.org/licenses/by/4.0/ This content is distributed under the terms of the Creative Commons Attribution 4.0 International license.

Afterward, we examined whether overexpressed OTOF inhibits replication-competent HIV-1 transmission in macrophages. A lentiviral vector expressing OTOF was transduced into primary MDMs. OTOF overexpression restricted R5-tropic HIV-1_AD8_ transmission in primary macrophages for 18 days (Fig. S4A and B), indicating that OTOF is a potential anti-HIV-1 factor in primary macrophages. Furthermore, we transduced lentiviral vectors overexpressing OTOF into stimulated CD4^+^ T lymphocytes and further infected these cells with HIV-1_NL4-3_ for 10 days. Data indicate that OTOF consistently restricted X4-tropic HIV-1 transmission in CD4^+^ T cells (Fig. S5A to C). In contrast, the proliferation of CD4^+^ T cells remained unaffected in the presence or absence of OTOF. Therefore, OTOF is possibly a novel anti-HIV-1 factor in immune cells.

10.1128/mbio.01738-22.4 FIG S4 OTOF overexpression inhibits HIV-1 transmission in macrophages. Lentiviral OTOF- or mock-expressing vectors that transduced MDMs were infected with HIV-1_AD8_ for 18 days. Viral production was measured using p24 ELISA at the indicated time points (A), and before infection, the aliquoted cells were lysed for Western blotting to assess OTOF and GAPDH protein levels using specific antibodies (B). Download FIG S4, PDF file, 0.2 MB. Copyright © 2022 Ding et al. 2022 Ding et al. https://creativecommons.org/licenses/by/4.0/ This content is distributed under the terms of the Creative Commons Attribution 4.0 International license.

10.1128/mbio.01738-22.5 FIG S5 OTOF overexpression inhibits HIV-1 transmission in CD4^+^ T cells. Lentiviral OTOF- or mock-expressing vectors that transduced stimulated CD4^+^ T cells were infected with 5 or 50 ng of HIV-1_NL4-3_ for 10 days. Viral production was measured via p24 ELISA at the indicated time points (A) and the cell proliferation levels were assessed using Cell Trace (unstimulated CD4^+^ T cells served as the technique control) (B). Before infection, the aliquoted cells were also lysed for Western blotting to assess OTOF and GAPDH protein levels using specific antibodies (C). Data are plotted as the means ± standard deviations from technical triplicates. All data are representative of three independent experiments. Download FIG S5, PDF file, 0.1 MB. Copyright © 2022 Ding et al. 2022 Ding et al. https://creativecommons.org/licenses/by/4.0/ This content is distributed under the terms of the Creative Commons Attribution 4.0 International license.

To examine whether OTOF has a broad antiviral activity, its activity against HIV-2, simian immunodeficiency virus (SIV), EIAV, and MLV was assessed in target cells. Interestingly, the ectopic expression of OTOF significantly suppressed only HIV-2 infection (Fig. 3B) and exerted a mild effect on SIV infection (Fig. 3C) but had no evident effect on EIAV (Fig. 3D) or MLV (Fig. 3E) infection in target cells. Thus, OTOF exerts an inhibitory effect on HIV-1, HIV-2, and SIV in target cells but not on EIAV and MLV (see below for discussion).

Because OTOF possesses an ectodomain comprising a FerB (a central domain B in proteins belonging to the ferlin family) domain, three C2 (protein kinase C conserved region 2) domains (including a Ca^2+^-dependent membrane-targeting module), and a low-complexity region, followed by a short membrane-spanning region and cytoplasmic tail (Fig. S6A), we generated OTOF mutants (Δ540–640, Δ720–820, and Δ1170–1250) based on previous studies (34, 35) and transfected the expression vectors encoding the wild-type or mutant OTOF into 293T cells. The absence of the C-terminal transmembrane region (Δ1170–1250) and low-complexity region (Δ540–640), but not the C2 region (Δ720–820), abolished the inhibitory effects of OTOF on HIV-1 infection (Fig. S6B and C); this indicates that the low-complexity domain is crucial for the antiviral activity of OTOF and the antiviral activity of OTOF relies on its translocation into the cell membrane. Furthermore, the cellular localization of OTOF and its mutants was explored in 293T cells using OTOF and its mutant fused with green fluorescent protein (GFP). As shown in Fig. S6D, the wild-type OTOF and the mutant OTOF with the loss of the C2 region (Δ720–820) that exerted inhibitory effects on HIV-1 consistently emitted clear fluorescent membrane signal. In contrast, the loss of C-terminal transmembrane region (Δ1170–1250) and loss of low-complexity region (Δ540–640) mutants of OTOF that lost their anti-HIV activities showed clear cytoplasmic patterns. Therefore, OTOF is likely a membrane protein that protects target cells from HIV-1 infection.

10.1128/mbio.01738-22.6 FIG S6 Anti-HIV activity of OTOF relies on targeting the cell membrane. (A) Schematic representation of OTOF domains. OTOF possesses an ectodomain comprising a FerB domain, three C2 regions, and a low-complexity region, followed by a short membrane-spanning region and a cytoplasmic tail. (B and C) 293T cells were transfected with a construct encoding FLAG-tagged OTOF or its mutants or a mock expression construct. Cells were infected with 100 ng of HIV-1_NL4-3.Luc.R−.E−_ (VSV-G) 24 h after transfection. At 24 h after infection, cells were lysed to measure luciferase reporter activity (B) and for Western blotting to assess exogenous protein and GAPDH levels using specific antibodies (C). ***, *P* < 0.001; **, *P* < 0.01 (two-tailed, unpaired Student’s *t* test). Data are plotted as the means ± standard errors of the means from three independent experiments. (D) 293T cells exogenously expressing the vector encoding OTOF-GFP or its mutants or the mock were visualized via microscopy. Scale bars, 10 μm. All Western blot data are representative of three independent experiments. Download FIG S6, PDF file, 0.3 MB. Copyright © 2022 Ding et al. 2022 Ding et al. https://creativecommons.org/licenses/by/4.0/ This content is distributed under the terms of the Creative Commons Attribution 4.0 International license.

### Ca^2+^ does not affect the inhibitory effect of OTOF on HIV-1.

**FIG 4:**
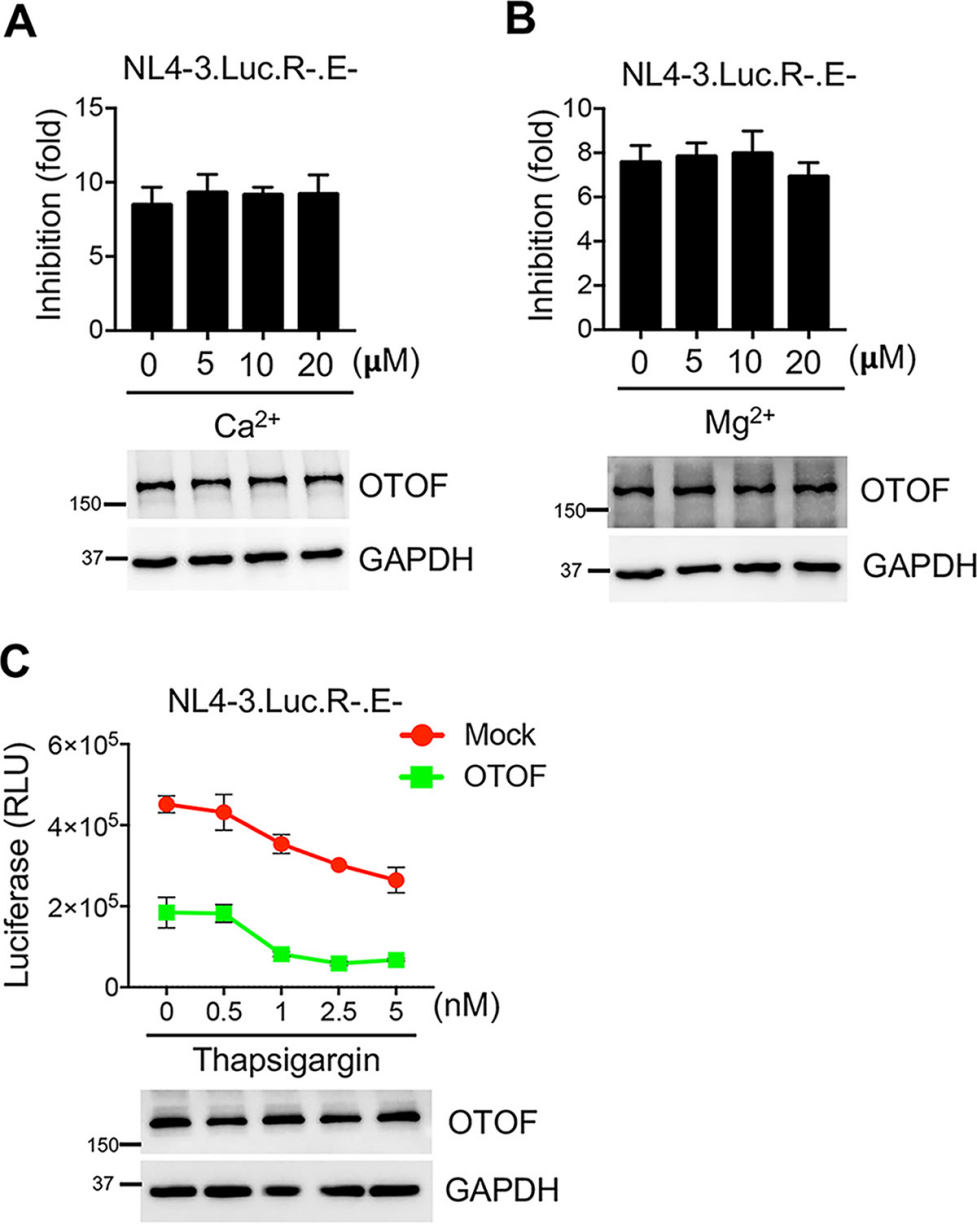
Antiviral activity of OTOF is independent of Ca^2+^. (A and B) 293T cells were transfected with a construct encoding FLAG-tagged OTOF or a mock expression construct. At 24 h after transfection, cells were infected with 100 ng of HIV-1_NL4-3.Luc.R−.E−_ (VSV-G) in the presence or absence of Ca^2+^ (A) or Mg^2+^ (B) at the indicated doses. At 24 h after infection, cells were lysed to measure luciferase reporter activity and for Western blotting to assess the levels of OTOF and GAPDH using specific antibodies. The inhibition of viral infection was accordingly calculated for OTOF and mock. Data are plotted as the means ± standard errors of the means from three independent experiments. (C) 293T cells were transfected with a construct encoding FLAG-tagged OTOF or a mock expression construct. At 24 h after transfection, cells were infected with 100 ng of HIV-1_NL4-3.Luc.R−.E−_ (VSV-G) in the presence or absence of thapsigargin at the indicated doses. At 24 h after infection, cells were lysed to measure luciferase reporter activity and for Western blotting to assess the levels of OTOF and GAPDH using specific antibodies. Data are plotted as the means ± standard deviations from three triplicates. All Western blot data are representative of three independent experiments. Numbers at left of blots are molecular masses in kilodaltons.

Because OTOF is a Ca^2+^ sensor at the auditory inner hair cell ribbon synapse (34), we investigated the role of Ca^2+^ in the anti-HIV-1 activity of OTOF. The anti-HIV-1 activity of OTOF was evaluated in the presence or absence of additional Ca^2+^. The inhibitory effect of OTOF was not evidently affected in the presence of Ca^2+^ at any dose used in the experiment (Fig. 4A). Similarly, the anti-HIV-1 activity of OTOF remained unaffected in the presence of Mg^2+^ (Fig. 4B), suggesting that the additional treatment with Ca^2+^ is insignificant in the anti-HIV-1 activity of OTOF. We used a selective and membrane-permeant Ca^2+^ chelator, thapsigargin, to assess the anti-HIV activity of OTOF. Ca^2+^ depletion did not significantly abolish the inhibitory effect of OTOF on HIV-1 (Fig. 4C). Thus, OTOF may not require Ca^2+^ to inhibit HIV-1 infection in target cells.

### OTOF restricts HIV-1 entry into target cells.

**FIG 5:**
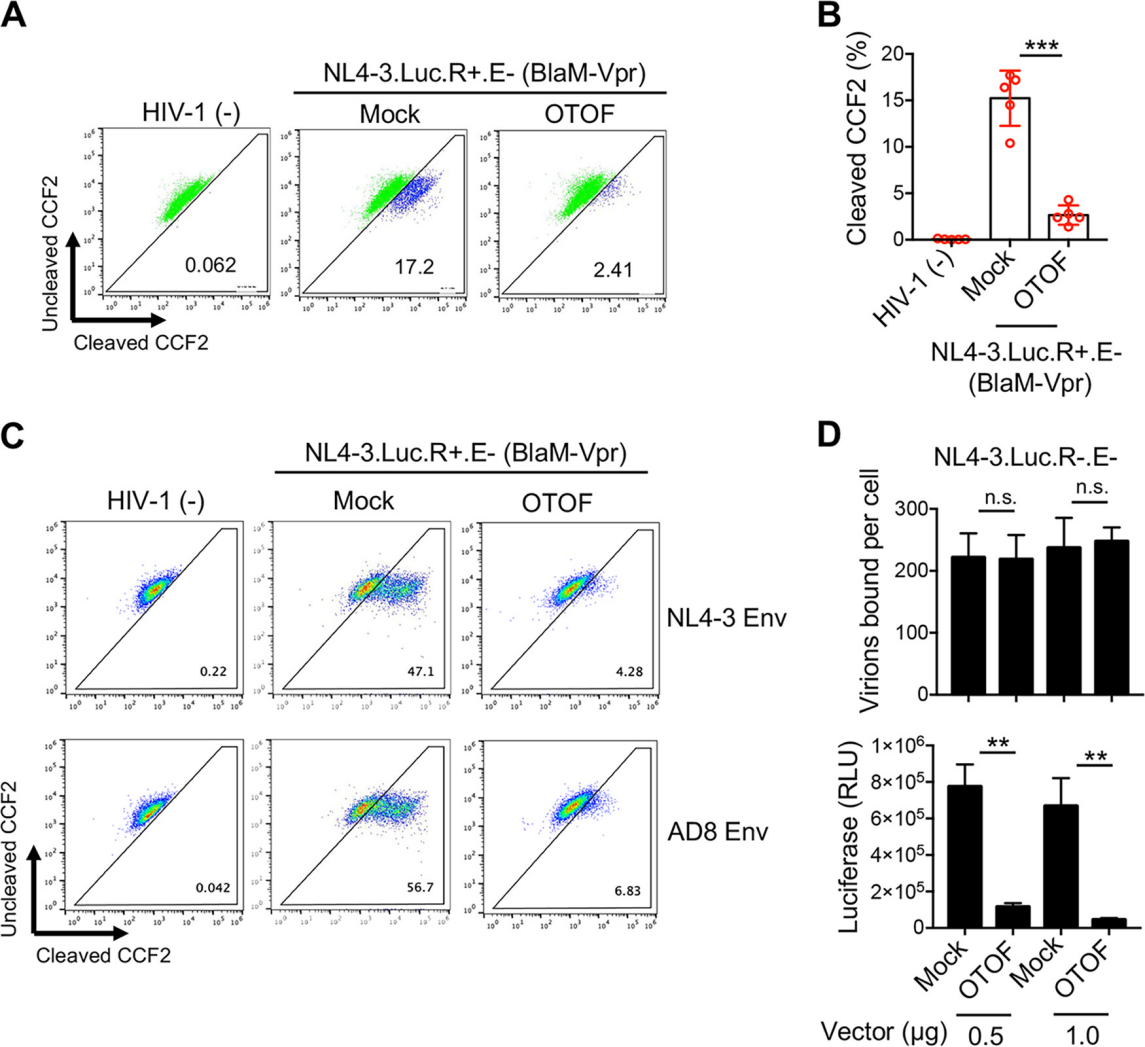
OTOF restricts HIV-1 entry into target cells. (A and B) 293T cells were transfected with a construct encoding FLAG-tagged OTOF or a mock expression construct. At 24 h after transfection, cells were infected with or without 100 ng of HIV-1_NL4-3.Luc.R+.E−_ (VSV-G) reporter virions incorporating the fusion protein BlaM-Vpr. Fluorescence-activated cell sorting dot plots are representative of five independent experiments (A). Data of five independent experiments are plotted as the means ± standard errors of the means from three independent experiments (B). (C) TZM-bl cells were transfected with a construct encoding FLAG-tagged OTOF or a mock expression construct. At 24 h after transfection, cells were infected with or without 100 ng of HIV-1_NL4-3.Luc.R+.E−_ (with NL4-3- or AD8-Env) reporter virions incorporating the fusion protein BlaM-Vpr. Representative fluorescence-activated cell sorting dot plots are presented from three independent experiments. (D) OTOF did not affect the ability of HIV-1 virions to attach to target cells. 293T cells were transfected with a construct encoding FLAG-tagged OTOF or a mock expression construct. At 24 h after transfection, cells were spinoculated with 500 ng of HIV-1_NL4-3.Luc.R−.E−_ (VSV-G) particles at 25°C for 2 h and washed with PBS twice to remove the input virus after spinoculation. Cells were further lysed for the p24 ELISA to quantify the bound viral particles (upper panel). In the parallel experiment, 293T cells were transfected with the same vectors of FLAG-tagged OTOF or a mock expression construct. At 24 h after transfection, cells were infected with 100 ng of HIV-1_NL4-3.Luc.R−.E−_ (VSV-G) for 24 h, and cells were lysed to measure luciferase reporter activity (lower panel). **, *P* < 0.01; ***, *P* < 0.001; n.s., not significant (two-tailed, unpaired Student’s *t* test). Data are plotted as the means ± standard errors of the means from three independent experiments.

To elucidate the mechanism through which OTOF inhibits HIV-1 infection, we analyzed the entry efficiency of HIV-1 particles into target cells in the presence of OTOF using the β-lactamase (BlaM)-fused Vpr entry assay (39). Using this assay, the entry of virions carrying the BlaM-Vpr fusion protein can be assessed by measuring the activity of the BlaM delivered to target cells. The HIV-1_NL4-3.Luc.R+.E−_ viruses (VSV-G) carrying the BlaM-Vpr reporter protein were used to infect 293T cells in the presence or absence of OTOF. The entry efficiency of HIV-1 was markedly reduced in the presence of OTOF compared with that of the control (Fig. 5A); this result was observed in five independent experiments (Fig. 5B). Moreover, we explored whether OTOF inhibits the entry of HIV-1 with authentic envelope proteins into target cells. As shown in Fig. 5C, OTOF was consistently found to inhibit the entry of either CXCR4- or CCR5-tropic HIV-1 virions into target cells. Thus, the entry of HIV-1 virions into target cells was substantially impaired by OTOF. Next, we investigated whether OTOF restricts the ability of HIV-1 to cross the cell membrane. Then, HIV-1 virion attachment assays were performed, which showed that the ability of HIV-1 to attach to target cells remained intact in the presence of OTOF (Fig. 5D, upper panel). However, OTOF still suppressed HIV-1 replication in target cells in a parallel experiment (Fig. 5D, lower panel). Thus, OTOF may interfere with the HIV-1 virion across the cell membrane, restricting HIV-1 infection in target cells.

## DISCUSSION

IFN is produced as the first line of defense in response to HIV-1 infection, and its antiviral properties exert widespread effects on the immune system. Type I IFN stimulates the expression of HIV-1 restriction factors, which limit virus replication. These restriction factors act at nearly every stage of the HIV-1 replicative cycle, from fusion with the cell membrane (IFITMs and Ch25h) and reverse transcription (SAMHD1 and APOBEC3s) to nuclear entry (MX2), translation (Schlafen 11 and Shiftless), and budding (tetherin) (18–29, 40). The effects of type I IFN on HIV-1 replication may depend on the timing of IFN-stimulated gene expression in various host cells. In this study, we demonstrated that IFN-α induces a novel antiviral factor—the membrane protein OTOF—to inhibit HIV-1 virion entry in macrophages and DCs. We first compared the gene expression profiles of PBMCs isolated from the blood of untreated patients with HIV-1 infection and healthy donors. OTOF expression was found to be highly induced in the PBMCs of untreated patients compared with those of healthy donors. Additionally, the IFN-α-inducible IFI27 level was lower than that of OTOF; thus, whether OTOF induction is caused by IFN-α stimulation should be explored further. Afterward, we treated primary CD4^+^ T cells, macrophages, and DCs with IFN-α and observed that OTOF was highly induced in these cells. Compared with IFN-α stimulation in macrophages and DCs, IFN-α exhibited a low level of OTOF induction in primary CD4^+^ T cells. OTOF is not expressed in certain host cell lines such as 293T, Jurkat, or HeLa cells. Hence, we studied the physiological antiviral activity of OTOF in myeloid cells. In the absence of OTOF, the resistance of IFN-α to HIV-1 infection was impaired in primary macrophages or DCs, indicating OTOF as a downstream weapon that IFN-α uses against HIV-1 infection in myeloid cell lineages. We further noted that depletion of OTOF did not affect the expression of *ISG-15* or the protein level of IFITM3, an IFN-α-induced anti-HIV factor inhibiting virion fusion with the cell membrane. Thus, the knockdown of OTOF does not affect the primary macrophage response to IFN stimulation.

Ectopic expression of OTOF inhibited HIV-1 and HIV-2 infections and mildly inhibited SIV infection, but not EIAV and MLV infection, in target cells. EIAV and MLV virions may target different microdomains of the cell membrane where OTOF is largely absent, or their fusion with the cell membrane requires different factors that OTOF cannot recognize to hinder virion penetration into the cell; this requires further investigation. Thus, OTOF does not exert a negative effect on EIAV and MLV. To further substantiate the important anti-HIV-1 activity of OTOF, we explored the activity of OTOF using replication-competent HIV-1. OTOF overexpression restricted the transmission of both replication-competent X4- and R5-tropic HIV-1 both in CD4^+^ T cells and in macrophages.

Because OTOF is a Ca^2+^ sensor (34), we examined the anti-HIV-1 activity of OTOF in the presence of Ca^2+^. Ca^2+^ did not affect the inhibitory effect of OTOF on HIV-1 infection compared with Mg^2+^, implying that the antiviral function of OTOF is not dependent on Ca^2+^. Furthermore, we investigated the molecular mechanisms that OTOF uses to inhibit HIV-1 infection. In the BlaM-Vpr-based viral entry assay, OTOF significantly impaired the entry of HIV-1 into target cells. Further evidence revealed that OTOF does not affect the attachment of HIV-1 virions to target cells; however, it may inhibit HIV-1 virion across the cell membrane. Taken together, we propose that OTOF might function as a fusion inhibitor to disturb the fusion of HIV-1 virions with the membrane of target cells, thereby inhibiting HIV virion entry. OTOF does not block virions from attaching to target cells. In this study, we did not elucidate the detailed mechanism through which OTOF inhibits HIV-1 entry. The following possibilities requires further investigation: OTOF hinders virion particles and the cell membrane from coming into close proximity to disturb the initial prefusion conformation, OTOF inhibits curvature induction mediated by loops of the viral fusion proteins, or OTOF tethers HIV-1 virions to the cell membrane to inhibit further endocytosis. Future studies focusing on OTOF-binding SNARE proteins may elucidate the mechanism through which OTOF inhibits HIV-1 entry into target cells (34). Furthermore, silencing *OTOF* partially relieved IFN-α-induced resistance to HIV-1 infection in macrophages or DCs. We speculate that additional IFN-stimulated factors that interfere with HIV-1 replication in macrophages remain to be discovered.

## MATERIALS AND METHODS

### Ethics statement.

The Research and Ethics Committee of The First Affiliated Hospital of China Medical University approved this study. All participants provided written informed consent before the commencement of the study in accordance with the National Health and Medical Research Council guidelines. The study protocol and informed consent forms were approved by the Institutional Review Board of China Medical University.

### Participants.

This study enrolled patients with HIV-1 infection who were or were not receiving combination ART (viral loads of <50 copies/mL) as well as HIV-1-negative healthy individuals. Blood samples were collected from these participants, and PBMCs were prepared via Ficoll-Hypaque density gradient centrifugation. The cells were cryopreserved in fetal calf serum supplemented with 10% dimethyl sulfoxide and stored in liquid nitrogen within 8 h of collection.

### Cells and culture reagents.

HeLa, 293T, TZM-bl, THP-1, and Jurkat cells were grown and maintained in Dulbecco’s modified Eagle’s medium (Gibco) or Roswell Park Memorial Institute Medium 1640 (Gibco). Both media were supplemented with 10% fetal bovine serum (FBS; Gibco), 100 U/mL penicillin, and 100 mg/mL streptomycin. Plasmids were transfected into 293T cells using Lipofectamine 2000 (Invitrogen) according to the manufacturer’s instructions. PBMCs obtained from healthy blood donors were purified via Ficoll-Hypaque gradient centrifugation. CD4^+^ T cells and monocytes were isolated from PBMCs via negative selection with human CD4^+^ T cells or a CD14-positive enrichment cocktail (StemCell Technologies). To stimulate CD4^+^ T cells, CD3/CD28 activator magnetic beads (Invitrogen) were added to the culture medium for 2 days in addition to interleukin-2 (IL-2) (50 U/mL; Biomol) according to the manufacturer’s instructions. Monocytes, MDMs, and MDDCs were isolated and cultured as described previously (41). MDMs were generated by stimulating monocytes with 10 ng/mL recombinant human granulocyte-macrophage colony-stimulating factor (GM-CSF; R&D) and 50 ng/mL recombinant human macrophage colony-stimulating factor (M-CSF; R&D) for 7 days. MDDCs were generated by incubating CD14-purified monocytes in Iscove’s modified Dulbecco’s medium (Gibco) supplemented with 10% FBS, 2 mM l-glutamine, 100 IU/mL penicillin, 100 mg/mL streptomycin, 10 mM HEPES, 1% nonessential amino acids, 1 mM sodium pyruvate, 10 ng/mL GM-CSF, and 50 ng/mL IL-4 (Miltenyi Biotec). On day 4, two-thirds of the culture medium was replaced with a fresh medium containing GM-CSF and IL-4. Immature MDDCs were harvested and used for experiments on day 6. Lipofectamine 3000 (Thermo Fisher) was used for siRNA delivery into MDMs or MDDCs according to the manufacturer’s instructions.

### Chemical reagents.

CaCl_2_, MgCl_2_, and thapsigargin were purchased from Sigma-Aldrich. IFN-α-2A (catalog no. 14276) was purchased from Sigma-Aldrich.

### Plasmids.

OTOF expression vectors were purchased from OriGene, or their open reading frames were *de novo* cloned into the pCMV-3Tag-2A (Addgene) vector. HIV-1 proviral vectors of NL4-3, NL4-3.Luc.R^−^.E^−^, and NL4-3.Luc.R^+^.E^−^ were obtained through the NIH AIDS reagent program. Eric Freed provided the HIV-1 proviral vectors of pNL-AD8, Xiaojun Wang provided the luciferase-expressing Env-deficient EIAV-luc reporter plasmid, and Guangxia Gao provided the Env-deficient, SIVmac239-Luc, and HIV-2Rod-Luc MLV-luc reporter plasmids as well as the Env expression vectors derived from NL4-3, AD8, 89.6, SF162, AE, and R3A (42). Recombinant lentiviruses for the shRNAs (catalog no. TRCN0000006984 and TRCN0000006982; open reading frame targeting) or a control (catalog no. SHC002; nonmammal targeting) were generated via the transient transfection of lentiviral shRNA vectors and translentiviral shRNA packaging plasmids (pMD2.D and psPAX2; Sigma) in 293T cells according to the manufacturer’s instructions (Sigma). The shRNA sequences were as follows: TRCN0000006984, 5′-CCGGCCTGTCTTTGGGAAGTCCTTTCTCGAGAAAGGACTTCCCAAAGACAGGTTTTT; TRCN0000006982, 5′-CCGGCCGCCCATCATTGTCATTGAACTCGAGTTCAATGACAATGATGGGCGGTTTTT; SHC002, 5′-CGGCAACAAGATGAAGAGCACCAACTCGAGTTGGTGCTCTTCATCTTGTTGTTTTTT.

### RNAi in THP-1 cells, MDMs, or MDDCs.

To achieve shRNA-mediated silencing of OTOF, an shRNA lentivirus was introduced to THP-1 cells and MDMs. Freshly isolated monocytes were briefly treated with VLP-Vpx and transduced with shRNA lentivirus particles (43, 44). After puromycin selection, the cells were infected with HIV-1 and washed twice with cold phosphate-buffered saline (PBS) to remove the input virus 6 h after infection. Stealth-grade siRNA (catalog no. HSS113923 and HSS190263) against OTOF and controls (catalog no. 12935112) were purchased from ThermoFisher. For siRNA-mediated silencing, MDMs and MDDCs were directly transfected with siRNA using Lipofectamine 3000 (ThermoFisher).

### Virion detection.

Enzyme-linked immunosorbent assay (ELISA) was used to measure p24 levels in culture supernatants according to the manufacturer’s instructions (ABL Corporation).

### Luciferase detection assay.

Luciferase activity was quantified in relative luminescence units in the cell lysates according to the manufacturer’s instructions (Promega). The TZM-bl reporter cell line was used for the quantitative analysis of HIV-1 infectivity using luciferase as the reporter, and the luminescence units of the background of TZM-bl cells were subtracted from each data point.

### Plasma HIV RNA measurement.

The total count of CD4^+^ T cells in whole blood was determined using the FACSCalibur flow cytometer (BD Biosciences, NJ, USA). The standard lyse/no-wash procedure was used with anti-CD4-fluorescein isothiocyanate (FITC)/CD8-phycoerythrin (PE)/CD3-peridinin chlorophyll protein (PerCP) as staining reagents and TruCount tubes (BD Biosciences, NJ, USA). TruCount control beads were used for quality control. The Roche Amplicor monitor standard assay (COBAS AmpliPrep/COBAS TaqMan HIV Test; Roche, Switzerland) was used to detect HIV RNA in plasma. The values were calculated according to the manufacturer’s reference standards. The limit of detection was set at 20 copies/mL.

### RNA-Seq analysis.

Freshly isolated PBMCs from five untreated patients and five healthy donors were lysed for total RNA extraction. A total of 3 μg RNA per sample was used as input material for RNA sample preparations. First, rRNA was removed using the Epicentre Ribo-Zero rRNA removal kit (Epicentre, USA), and the rRNA-free residue was cleaned via ethanol precipitation. Subsequently, sequencing libraries were generated using the rRNA-depleted RNA via NEBNext Ultra directional RNA library prep kit for Illumina (New England Biolabs [NEB], USA) following the manufacturer’s recommendations. Briefly, fragmentation was performed using divalent cations under elevated temperature in NEBNext first-strand synthesis reaction buffer (5×). First-strand cDNA was synthesized using a random hexamer primer and Moloney murine leukemia virus (M-MuLV) reverse transcriptase (RNase H). Second-strand cDNA was then synthesized using DNA polymerase I and RNase H. In the reaction buffer, dTTPs were replaced with dUTP. The remaining overhangs were converted into blunt ends via exonuclease/polymerase activity. After adenylation of the 3′ ends of DNA fragments, the NEBNext adaptor with a hairpin loop structure was ligated for hybridization. To select 250- to 300-bp cDNA fragments, the library fragments were purified using the AMPure XP system (Beckman Coulter, Beverly, MA, USA). Then, 3 μL User enzyme (NEB, USA) was incubated with size-selected, adaptor-ligated cDNA at 37°C for 15 min followed by 5 min at 95°C before PCR. Subsequently, PCR was performed with Phusion high-fidelity DNA polymerase, Universal PCR primers, and index (X) primer. Finally, the PCR products were purified (AMPure XP system); library quality was assessed using the Agilent Bioanalyzer 2100 system. Clustering of the index-coded samples was performed on a cBot cluster generation system using the TruSeq PE cluster kit v3-cBot-HS (Illumina) according to the manufacturer’s instructions. Following cluster generation, the libraries were sequenced on an Illumina HiSeq 4000 platform, and 150-bp paired-end reads were generated. Raw data (raw reads) of FASTQ format were first processed through in-house Perl scripts. In this step, clean data (clean reads) were obtained by removing reads containing the adaptor sequence, reads containing ploy-N, and low-quality reads from raw data. Simultaneously, Q20, Q30, and GC content of the clean data were measured. All downstream analyses were performed using clean data with high quality. These cleaned reads were then mapped to the reference genome from the ENSEMBL database (http://www.ensembl.org/) using the TopHat2 (v. 2.0.14) program (45). Cufflinks (v. 2.1.1) was subsequently used to assemble the whole transcriptome and identify all possible transcripts (46). Transcript abundances (e.g., fragments per kilobase of transcript per million fragments mapped) were estimated, and differential analysis was performed using the cuffdiff command (47, 48). The mapping results in BAM format were converted to the tiled data file (TDF) format using Integrative Genomics Viewer (IGV) tools for visualization using IGV (49).

### Membrane-associating protein isolation.

Membrane-associating proteins were isolated according to the manufacturer’s instructions (Mem-PER Plus membrane protein extraction kit; Invitrogen), as described previously (41). Briefly, cells were harvested from the cell suspension after centrifugation at 300 × *g* for 5 min. The obtained cell pellets were washed with 3 mL of cell wash solution and centrifuged at 300 × *g* for 5 min. After removing the supernatants, the cell pellets were resuspended in 1.5 mL of cell wash solution and transferred to a new tube. Then, they were centrifuged at 300 × *g* for 5 min and the supernatants were discarded. The resultant cell pellets were added to 0.75 mL of permeabilization buffer to obtain a homogeneous cell suspension and incubated for 10 min at 4°C. The permeabilized cells were centrifuged for 15 min at 16,000 × *g*, and the resultant supernatants containing cytosolic protein were removed and transferred to a new tube for detection. The pellets were added to 0.5 mL solubilization buffer and resuspended by pipetting up and down; then, they were incubated for 30 min at 4°C with constant mixing. The resultant solution in the tubes was centrifuged at 16,000 × *g* for 15 min at 4°C. The supernatants containing the solubilized membrane and membrane-associated proteins were collected into fresh tubes for detection. The protein was subjected to sodium dodecyl sulfate–polyacrylamide gel electrophoresis and further assessed via Western blotting using specific antibodies.

### Viral infectivity measurement.

The levels of p24 in culture supernatants were measured using ELISA (ABL Corporation). The infectivity of HIV-1 in the culture supernatants was measured by infecting TZM-bl reporter cells with the virus. Then, viral infectivity was quantified using luciferase as the reporter. The luciferase activity in a cell lysate is quantified in relative luminescence units (Promega). Furthermore, the background luminescence of the control (uninfected) TZM-bl cells was subtracted from each data point.

### BlaM-Vpr-based viral entry assay.

HIV-1 particles incorporating a fusion protein between Vpr and BlaM reporter protein were produced by cotransfection of NL4-3.Luc.R^+^.E^−^ with an expression vector encoding BlaM-Vpr. The viruses produced were quantified using p24 ELISA, and target cells were incubated with 100 ng of p24 viruses at 37°C for 4 h to allow viral entry. After washing three times with Hanks’ balanced salt solution (HBSS; ThermoFisher), cells were resuspended and loaded with 1 μM CCF2-AM dye (ThermoFisher), a fluorescent substrate for BlaM, in HBSS containing 1 mg/mL Pluronic F-127 surfactant (ThermoFisher) and 0.001% acetic acid for 1 h at room temperature; then, they were washed twice with HBSS. The BlaM reaction, which corresponds to the cleavage of intracellular CCF2 dye by BlaM-Vpr, was performed for 14 h at room temperature in HBSS supplemented with 10% FBS. Cells were washed three times with PBS and fixed in a 1.2% solution of paraformaldehyde. The fluorescence was monitored at 520 and 447 nm via flow cytometry using Sony ID7000 cytometry.

### HIV-1 attachment assay.

The attachment of the viral particles to the cell membrane was assayed (50, 51). In brief, 3 × 10^5^ TZM-bl cells or 2 × 10^5^ stimulated CD4^+^ T cells were spinoculated with HIV-1 at 1,200 × *g* at 25°C for 2 h. Then, the infected cells were washed with cold medium five times to remove the unbound viral particles. Next, the cells were lysed with 0.5% Triton X-100 to quantify the cell-associated p24gag using ELISA. Virion equivalents were determined by assuming an average of 1,500 p24gag molecules per HIV-1 particle, i.e., 15,800 viral particles per pg of p24gag.

### Western blotting and antibodies.

Standard Western blotting was performed to detect cellular proteins using various antibodies, including monoclonal mouse anti-OTOF (Santa Cruz; C-12; catalog no. sc-271092; 1:1,000 dilution), rabbit anti-glyceraldehyde-3-phosphate dehydrogenase (anti-GAPDH) (ThermoFisher, catalog no. PA1-987; 1:1,000 dilution), mouse monoclonal anti-FLAG (Sigma; catalog no. F1804; 1:1,000 dilution), and mouse Ig-horseradish peroxidase (HRP) (Abcam; catalog no. 6789; 1:5,000 dilution).

### Microscopy.

The cells were photographed using the Zeiss LSM 980 with the Airyscan 2 imaging system.

### Quantitative PCR.

Total RNA was extracted from cells using TRIzol (Invitrogen) according to the manufacturer’s instructions. The obtained RNA was dissolved in 100 μL of diethyl pyrocarbonate (DPEC)-H_2_O, and 1 μg of the purified RNA was treated with DNase I (amplification grade; Invitrogen) for 10 to 15 min at room temperature according to the manufacturer’s instructions. The RNA was immediately primed with oligo(dT) and reverse transcribed using Superscript III reverse transcriptase (Invitrogen). Real-time PCR analysis was performed using the 2^−ΔΔ^*^CT^* method. The results were normalized against the amplification results for the internal control (GAPDH). Table S4 in the supplemental material shows the primers used in this study.

10.1128/mbio.01738-22.10 TABLE S4 Sequences of primers used in this study. Download Table S4, PDF file, 0.01 MB. Copyright © 2022 Ding et al. 2022 Ding et al. https://creativecommons.org/licenses/by/4.0/ This content is distributed under the terms of the Creative Commons Attribution 4.0 International license.

### Statistical analysis.

Statistical analysis was performed using Prism 6.0 (GraphPad Software). Unless otherwise specified, unpaired two-tailed Student’s *t* tests were used for between-group statistical comparisons.

### Data availability.

RNA-Seq data are accessible through the Sequence Read Archive (https://www.ncbi.nlm.nih.gov/bioproject) with the reference number PRJNA853066. Data supporting the findings of this study are available within this article and the supplemental material files as well as from the corresponding authors upon reasonable request.
