# Supplementary figures and images for: Membrane Protein OTOF Is a Type I Interferon-Induced Entry Inhibitor of HIV-1 in Macrophages
Source: mBio. 2022 Jul 18;13(4):e01738-22. doi: 10.1128/mbio.01738-22 (PMC9426595; doi:10.1128/mbio.01738-22)

# Figure S1

**A**

PBMCs

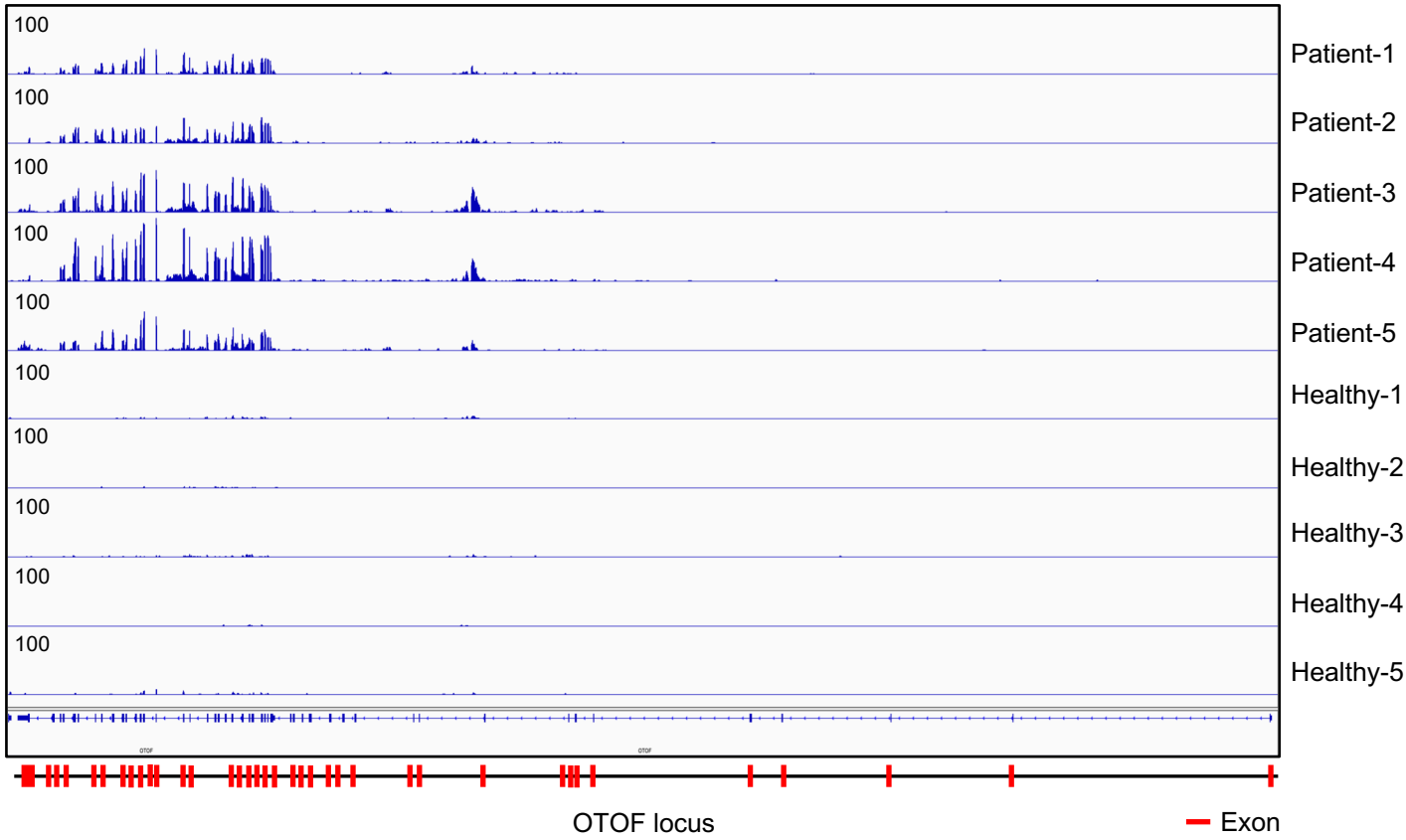**B**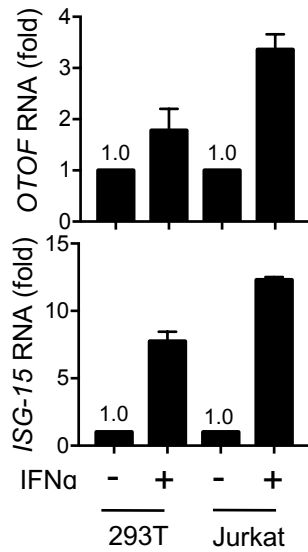**C**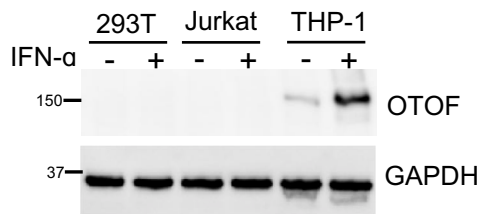

Supplement: FIG S1 [file mbio.01738-22-s0001.pdf]

# Figure S2

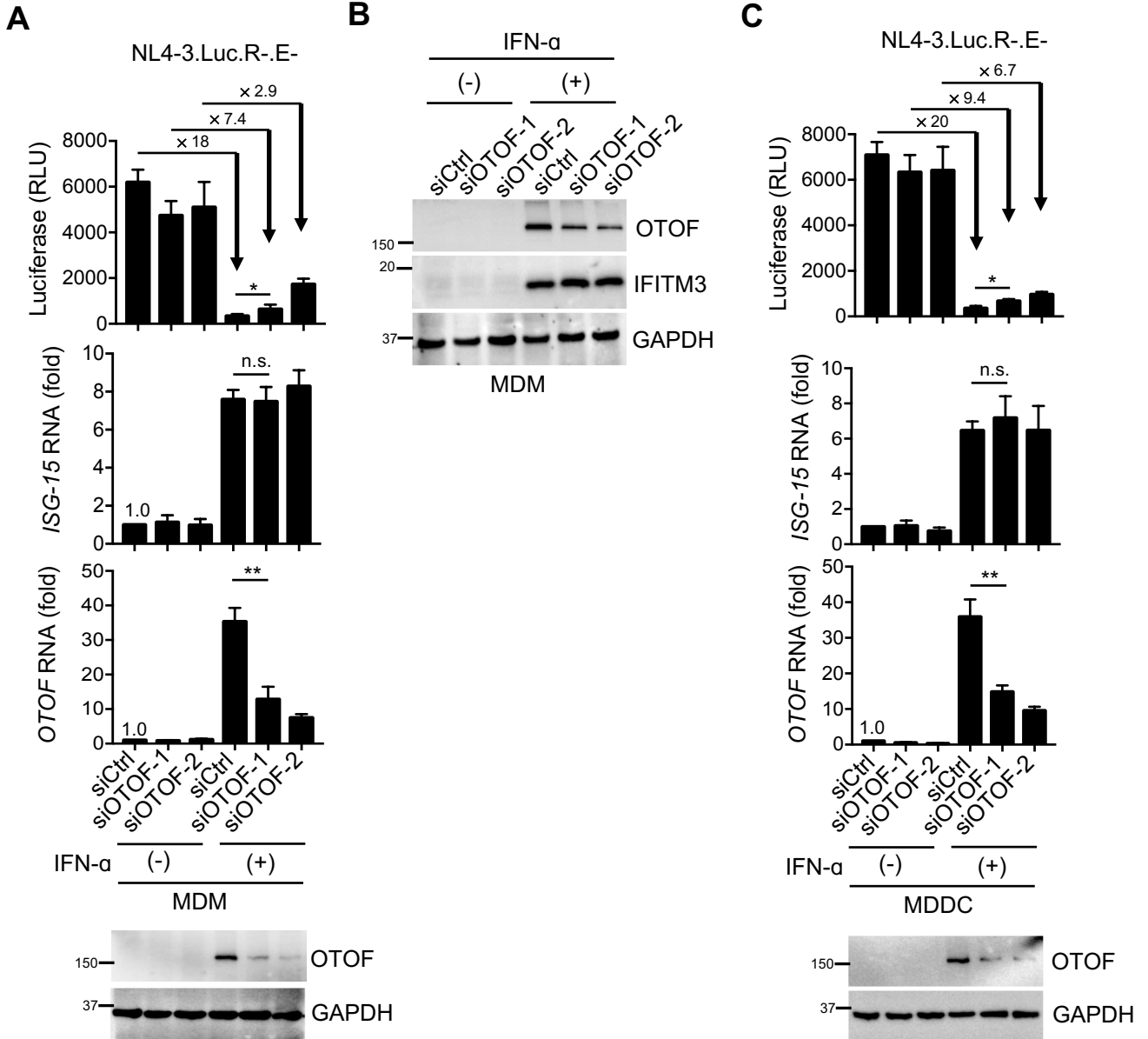

Supplement: FIG S2 [file mbio.01738-22-s0002.pdf]

# Figure S3

**A**

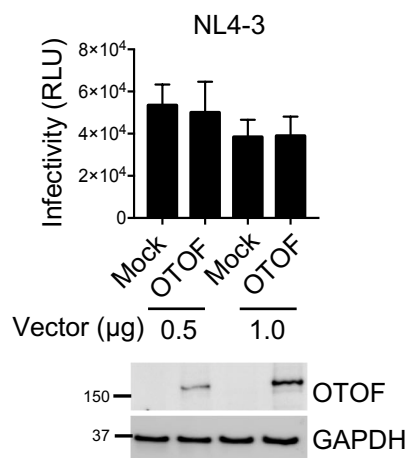

**B**

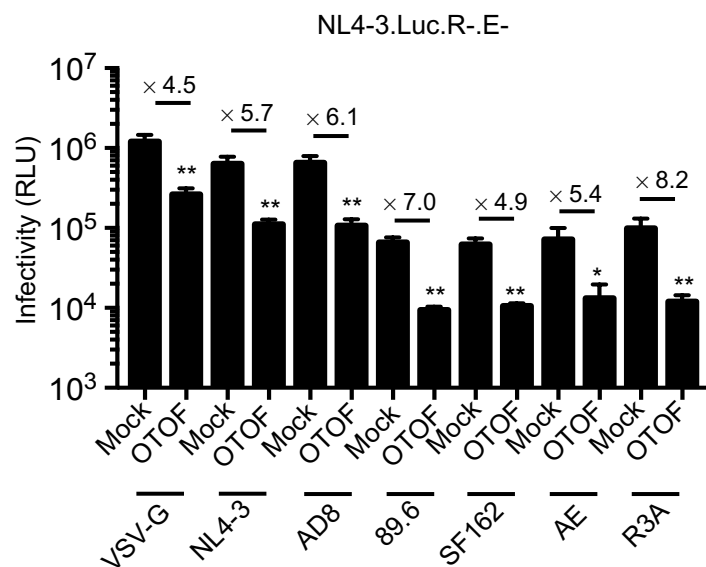

Supplement: FIG S3 [file mbio.01738-22-s0003.pdf]

# Figure S4

**A**

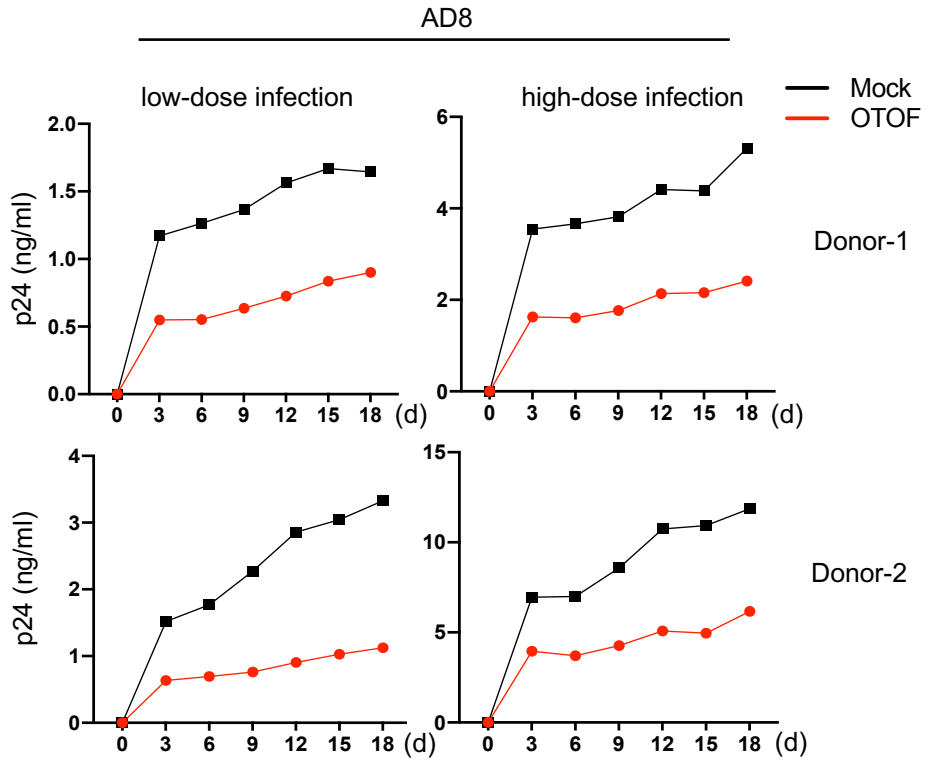

**B**

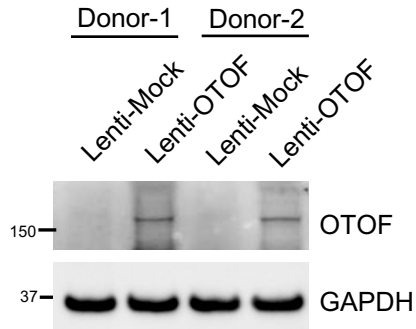

Supplement: FIG S4 [file mbio.01738-22-s0004.pdf]

**Figure S5**

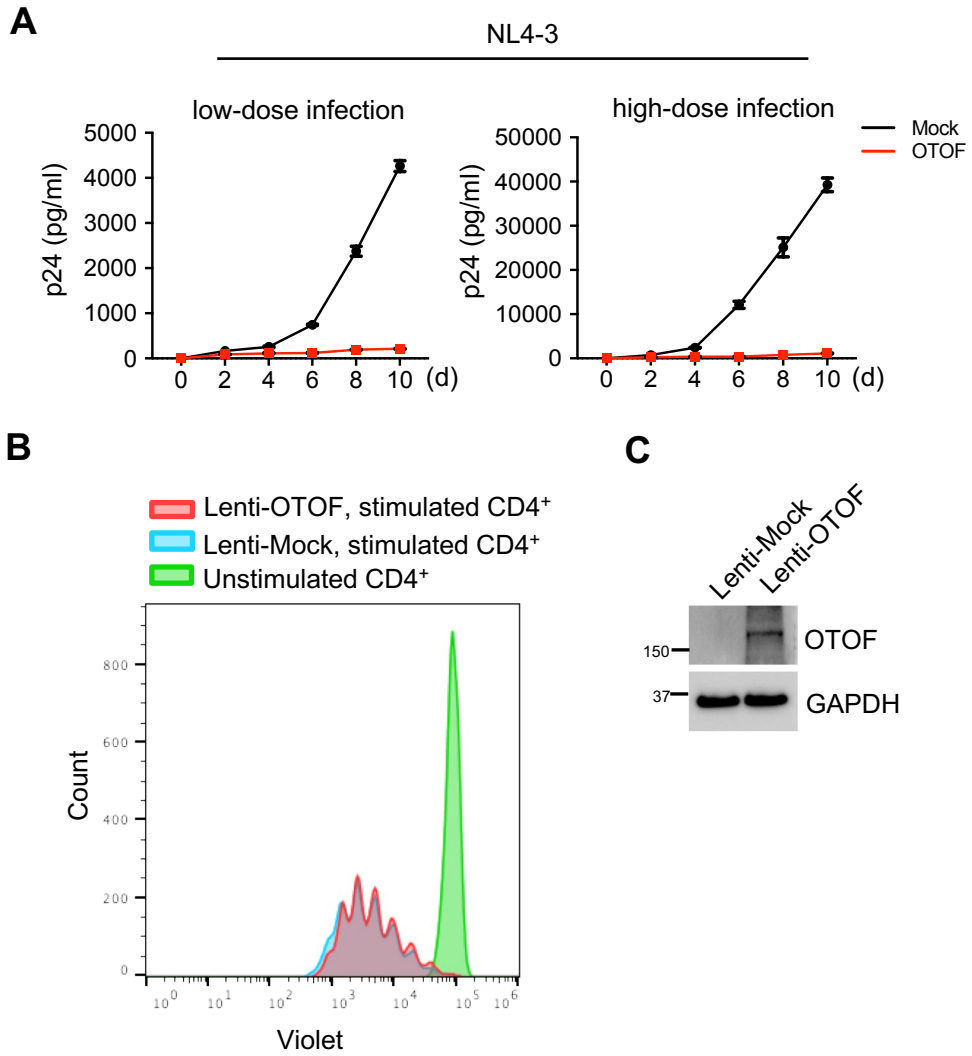

Supplement: FIG S5 [file mbio.01738-22-s0005.pdf]

# Figure S6

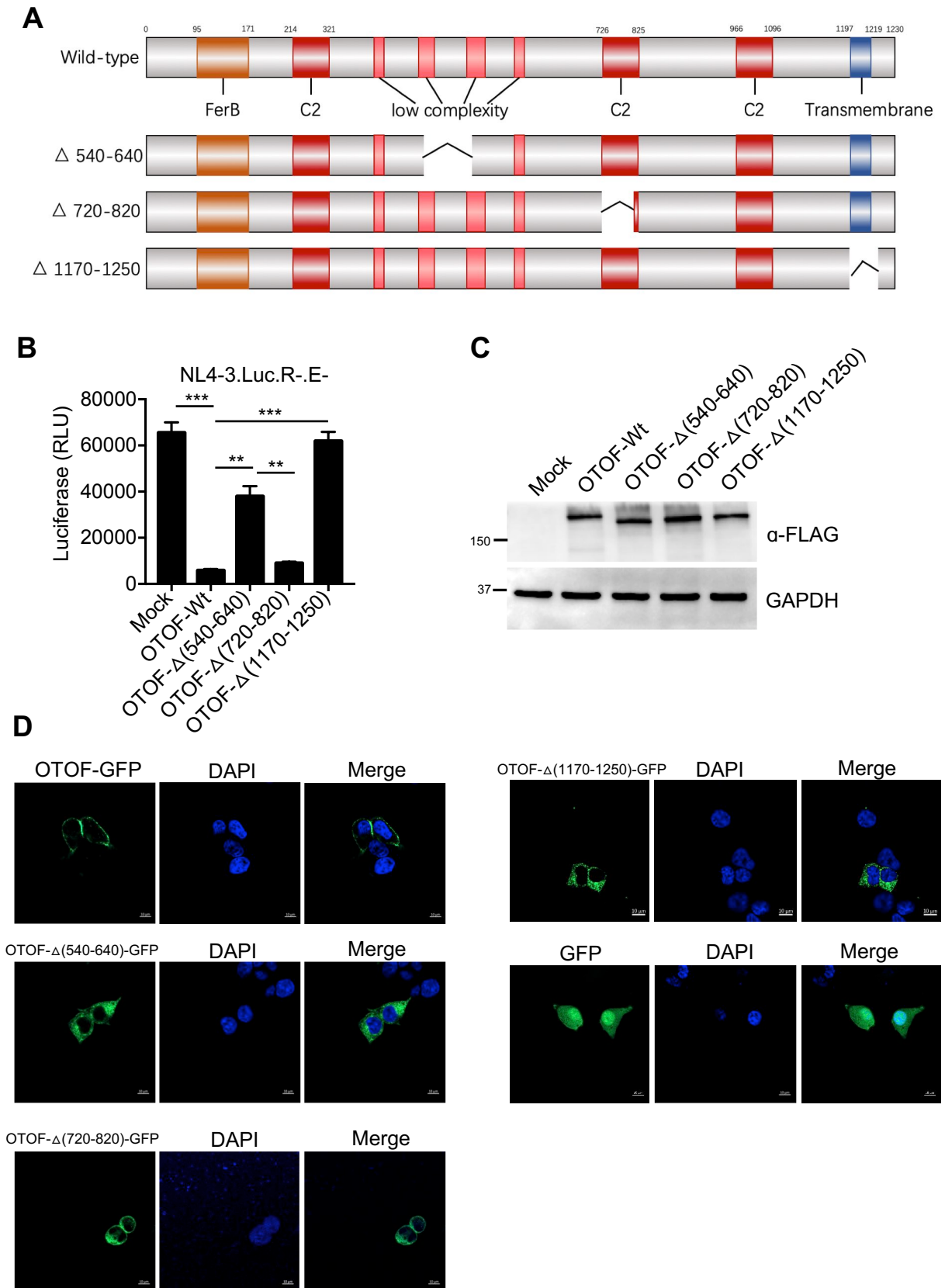

Supplement: FIG S6 [file mbio.01738-22-s0006.pdf]
