# Supplementary material for: Membrane Protein OTOF Is a Type I Interferon-Induced Entry Inhibitor of HIV-1 in Macrophages
Source: mBio. 2022 Jul 18;13(4):e01738-22. doi: 10.1128/mbio.01738-22 (PMC9426595; doi:10.1128/mbio.01738-22)
Supplement: TABLE S1 [file mbio.01738-22-s0007.pdf]

Table S1. The fold change (>10-fold) of gene expression between untreated patients and healthy donors.

Protein symbol	Fold change between HIV-1(+) and HIV(-)
OTOF	43.4
IFI27	29.6
SIGLEC1	15.4
CYP2F1	13.6
SERPING1	12.4
PRKCG	11.5
TMEM155	10.1
