# Supplementary material for: Membrane Protein OTOF Is a Type I Interferon-Induced Entry Inhibitor of HIV-1 in Macrophages
Source: mBio. 2022 Jul 18;13(4):e01738-22. doi: 10.1128/mbio.01738-22 (PMC9426595; doi:10.1128/mbio.01738-22)
Supplement: TABLE S2 [file mbio.01738-22-s0008.pdf]

Table S2. Backgrounds of untreated HIV-1-infected individuals in this study.

PID	Sex	Age	CD4 (cells/µL)	VL (copies/mL)
300029	Male	41	472	35000
300129	Male	40	267	51000
300163	Male	44	460	15800
300165	Male	46	360	15900
300168	Male	47	518	724
300173	Male	27	729	31400
300175	Male	27	315	9690
300183	Male	28	129	30000
300188	Male	41	177	127000
300210	Male	26	773	12100
300219	Male	47	376	5396
300222	Male	27	565	7280
300229	Male	26	352	4981
300237	Male	28	650	8420
300267	Male	48	36	444900
300280	Male	26	232	70300
300304	Male	29	389	84400
300308	Male	35	784	10500
300310	Male	28	700	22914
300312	Male	30	1234	23697
300328	Male	29	25	240000
300380	Male	21	365	830
300504	Male	40	212	76000
300507	Male	47	242	117000
300531	Male	48	898	21000
300608	Male	30	410	12400
