# Supplementary material for: Membrane Protein OTOF Is a Type I Interferon-Induced Entry Inhibitor of HIV-1 in Macrophages
Source: mBio. 2022 Jul 18;13(4):e01738-22. doi: 10.1128/mbio.01738-22 (PMC9426595; doi:10.1128/mbio.01738-22)
Supplement: TABLE S3 [file mbio.01738-22-s0009.pdf]

Table S3. Backgrounds of ART-treated HIV-1-infected individuals in this study.

PID	Sex	Age	CD4 (cells/μL)	VL (copies/mL)	Treatment
300064	Male	46	534	20.4	D4T+3TC+NVP
300185	Male	39	356	0	TDF+3TC+EFV
300189	Male	25	600	0	AZT+3TC+NVP
300229	Male	34	494	0	AZT+3TC+NVP
300539	Male	39	470	1	AZT+3TC+NVP
300576	Male	46	641	0	AZT+3TC+NVP
300823	Male	31	483	0	AZT+3TC+NVP
300975	Female	34	1047	0	AZT+3TC+EFV
300996	Male	36	551	0	AZT+3TC+NVP
301006	Male	56	646	0	TDF+3TC+EFV
301413	Male	42	480	0	TDF+3TC+EFV
301461	Male	31	617	0	TDF+3TC+EFV
301996	Male	39	725	0	TDF+3TC+EFV
303602	Male	33	294	0	TDF+3TC+EFV
303619	Male	57	439	0	TDF+3TC+EFV
303656	Male	26	627	0	AZT+3TC+NVP
303691	Male	36	495	0	AZT+3TC+NVP
303693	Male	28	502	0	AZT+3TC+NVP
320842	Male	41	466	10.5	TDF+3TC+EFV
322922	Male	47	580	0	AZT+3TC+NVP
