# Supplementary material for: Membrane Protein OTOF Is a Type I Interferon-Induced Entry Inhibitor of HIV-1 in Macrophages
Source: mBio. 2022 Jul 18;13(4):e01738-22. doi: 10.1128/mbio.01738-22 (PMC9426595; doi:10.1128/mbio.01738-22)
Supplement: TABLE S4 [file mbio.01738-22-s0010.pdf]

Table S4. Primer sequences used in this study.

Gene	Sequences
GAPDH for	5′- AATGACCCCTTCATTGAC
GAPDH rev	5′-TCCACGACGTACTCAGCGC
OTOF for	5′-GGGGCCTACGGGACCTAAA
OTOF rev	5′-AATTGTGGATCAGGGACGACT
ISG-15 for	5′-CGCAGATCACCCAGAAGATCG
ISG-15 rev	5′-TTCGTCGCATTTGTCCACCA
